# Supplementary material for: Conformation Selection by ATP-competitive Inhibitors and Allosteric Communication in ERK2
Source: bioRxiv. 2023 Sep 12:2023.09.12.557258. Preprint. [Version 1] doi: 10.1101/2023.09.12.557258 (PMC10515847; doi:10.1101/2023.09.12.557258)
Supplement: Supplementary file 1 [file media-1.pdf]

SUPPLEMENTAL MATERIALS

Anderson et al., "Conformation Selection by ATP-competitive Inhibitors and Allosteric Communication in ERK2"

SUPPLEMENTAL FIGURES

Figure S1. Structural features of ERK2.
Figure S2. Proteolytic peptides analyzed by HDX-MS
Figure S3. Structural map of regions showing HDX responses to inhibitor binding.
Figure S4. HDX time courses of regions with comparable responses to different inhibitors.
Figure S5. HDX time courses of differential responses to inhibitor binding.
Figure S6. Differential HDX responses to new inhibitors surveyed in this study.
Figure S7. Chemical shift changes induced by inhibitor binding to 2P-ERK2.
Figure S8. HDX time courses responsive to ATG017 binding.
Figure S9. Interactions of L16 and helix αL16 with N-lobe elements.
Figure S10. Crystal contacts with the activation loop in X-ray structures of ERK2

SUPPLEMENTAL TABLE

Table S1. X-ray data collection and refinement parameters

SUPPLEMENTAL DATAFILES

Datafile S1. Deuterium uptake time courses for all HDX-MS experiments (Excel format)
Datafile S2. NMR chemical shifts and perturbations by selected inhibitors (Excel format)
Datafile S3. X-ray coordinates for 2PERK2_Inhibitor#8 (PDB format)
Datafile S4. X-ray coordinates for 2PERK2_Inhibitor#16 (PDB format)

Figure S1

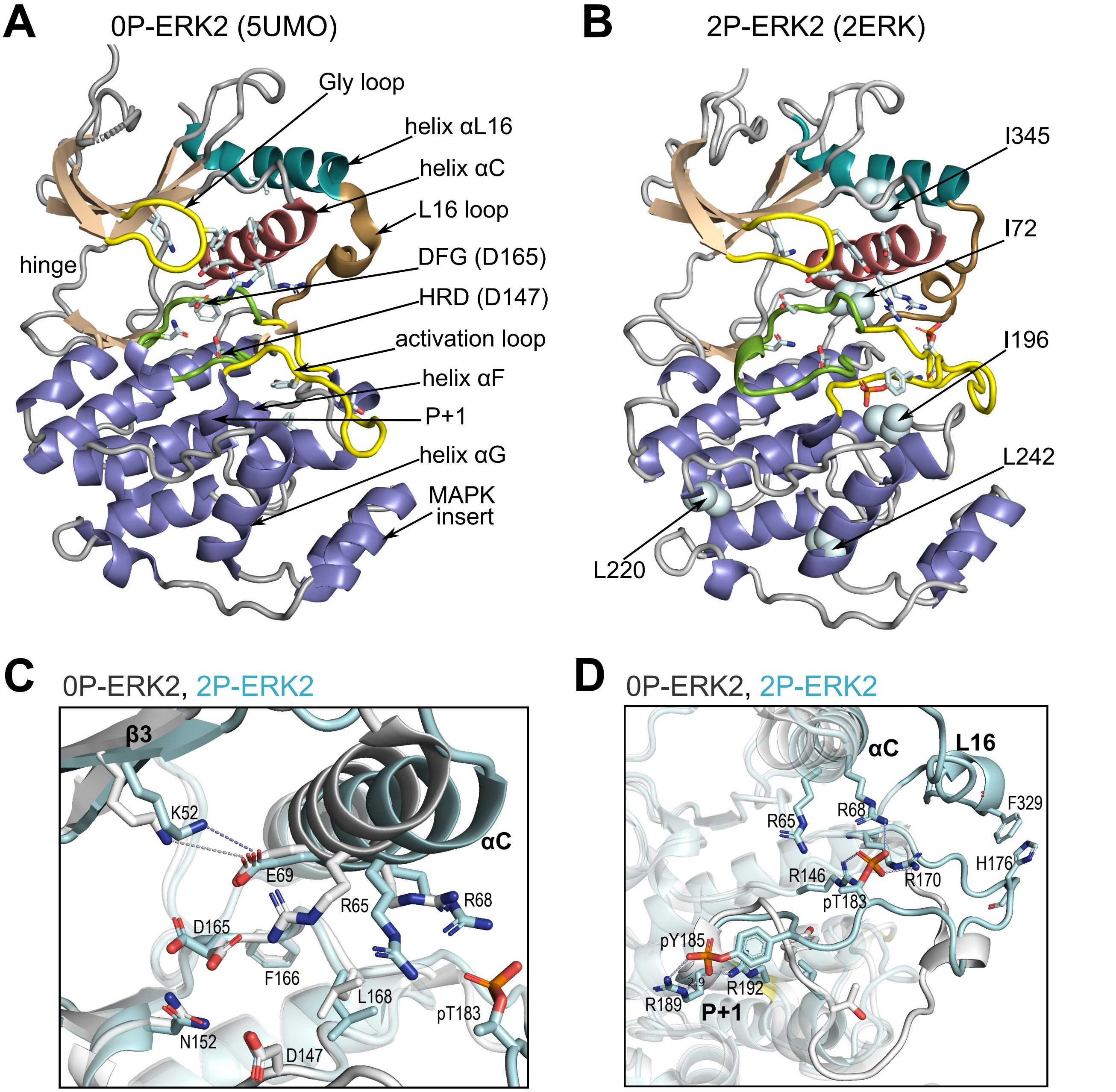

Figure S1. Structural features of ERK2. (A,B) X-ray structures of **(A)** 0P-ERK2 (PDBID:5UMO) and **(B)** 2P-ERK2 (PDBID:2ERK) apoenzymes. Panel **A** labels conserved motifs in ERK2 common to protein kinases. Panel **B** labels residues that illustrate L⇌R exchange (I72, L220, L242; Figs. 1 and 6), and key chemical shift perturbations (I196, I345; **Fig. 7**). **(C)** Structural superposition of 0P-ERK2 (white) and 2P-ERK2 (pale blue), illustrating overlapping positions of active site residues and an outward shift of helix αC upon dual phosphorylation. **(D)** Superposition of 0P-ERK2 and 2P-ERK2, illustrating conformational differences in the activation loop. In 2P-ERK2, pT183 and pY185 form ion pairs with multiple Arg residues, while the L16 loop folds into a 3/10 helix with side chain interactions to the activation loop. Structures were superpositioned by aligning Cα atoms within the C-terminal domain (residues 109-141, 205-245, 272-310).

Figure S2

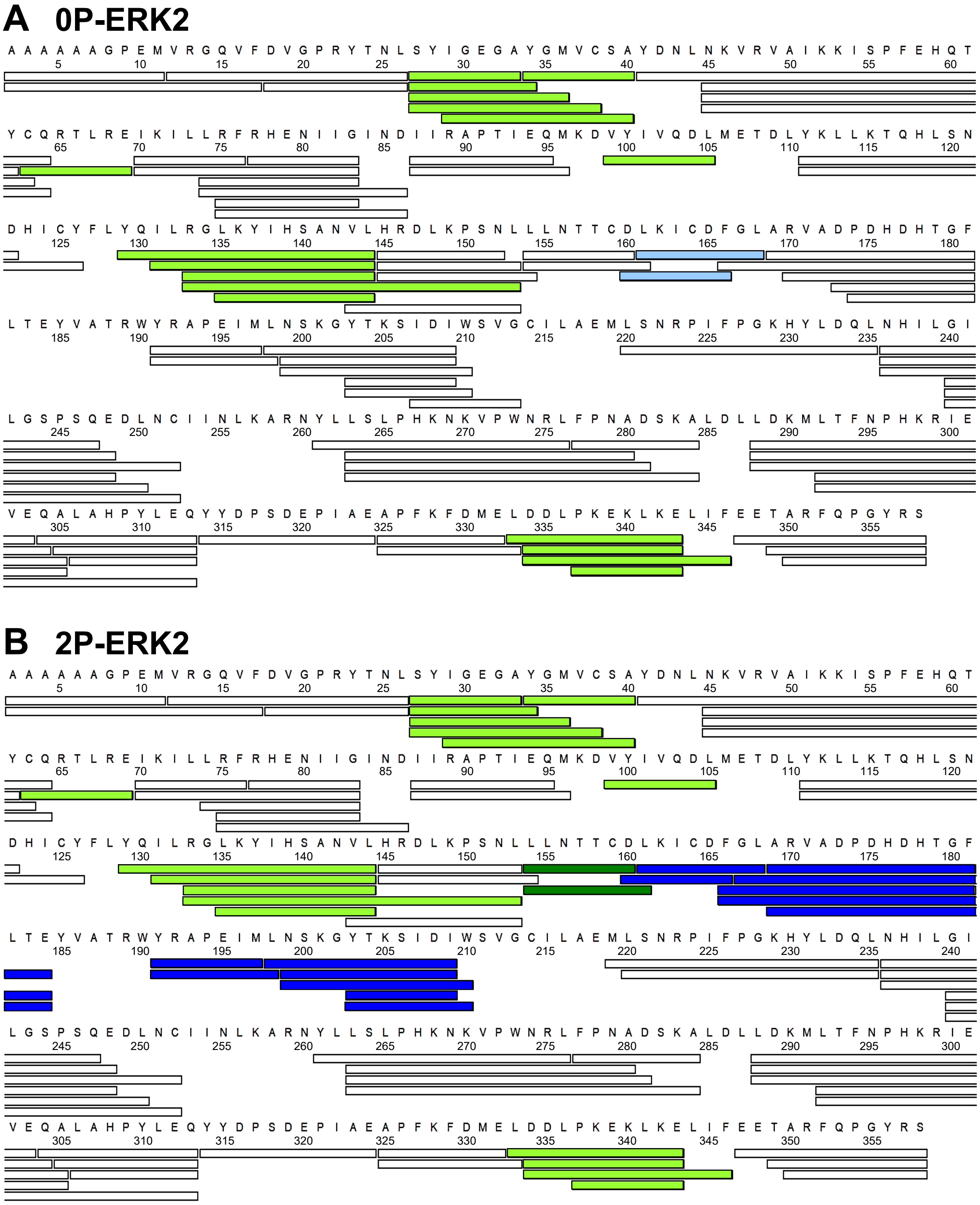

Figure S2. Proteolytic peptides analyzed by HDX-MS. Peptides produced by pepsin digestion yielded 90% and 91% coverage of exchangeable amides in **(A)** 0P-ERK2 and **(B)** 2P-ERK2, respectively. Colors indicate regions where ligand binding alters HDX uptake. Light green indicates peptides where binding of VTX11e, BVD523, and GDC0994 induce a similar degree of HDX protection (i.e., decreased deuterium uptake) in both 0P-ERK2 and 2P-ERK2. Deep green indicates peptides where all inhibitors induce a similar degree of HDX protection, but only in 2P-ERK2. Deep blue indicates peptides where VTX11e and BVD523 induce greater HDX protection compared to GDC0994 in 2P-ERK2. Light blue indicates peptides that show HDX protection by all inhibitors around the DFG motif in 0P-ERK2, but to a lower amount compared to BVD523 or VTX11e in 2P-ERK2.

Figure S3

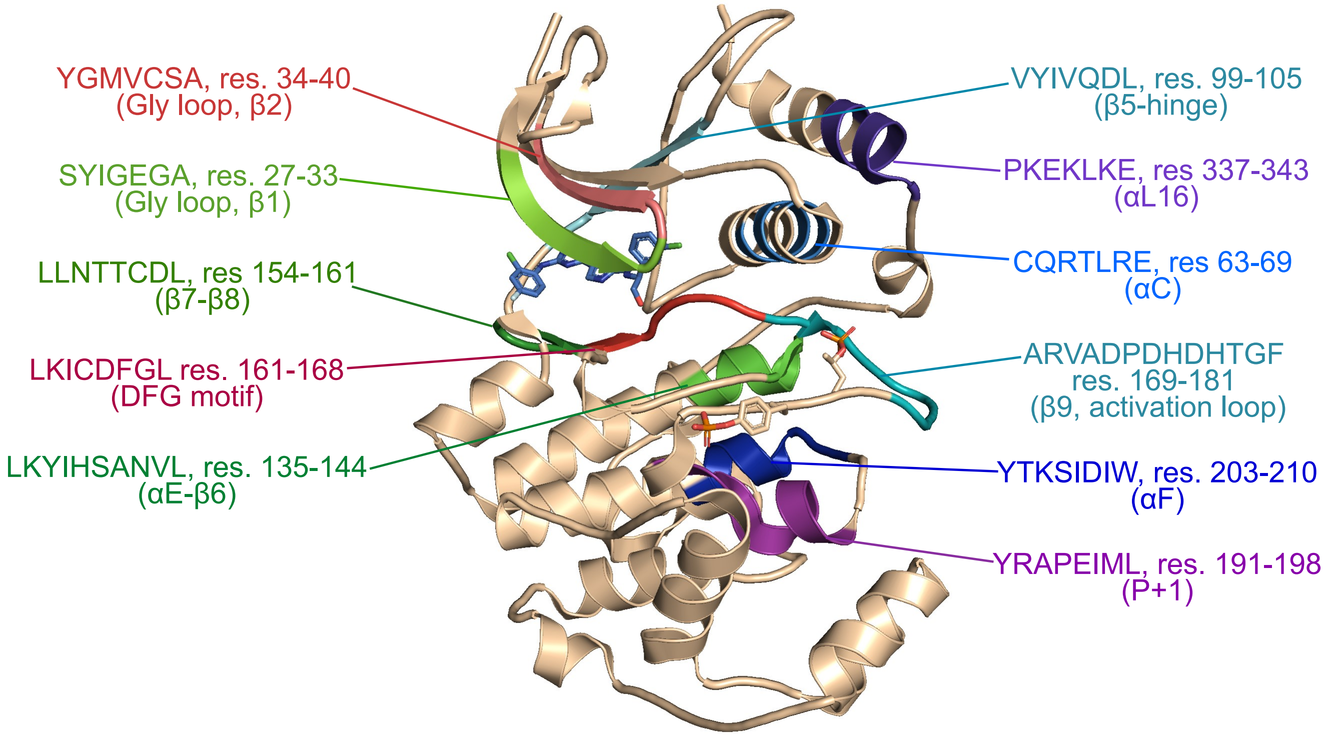

Figure S3. Structural map of regions showing HDX responses to inhibitor binding. Structure of 2P-ERK2 (PDBID:6OPK), indicating the locations of peptides where binding of VTX11e, BVD523 or GDC0994 alter HDX behavior, as highlighted in **Fig. S2**.

Figure S4

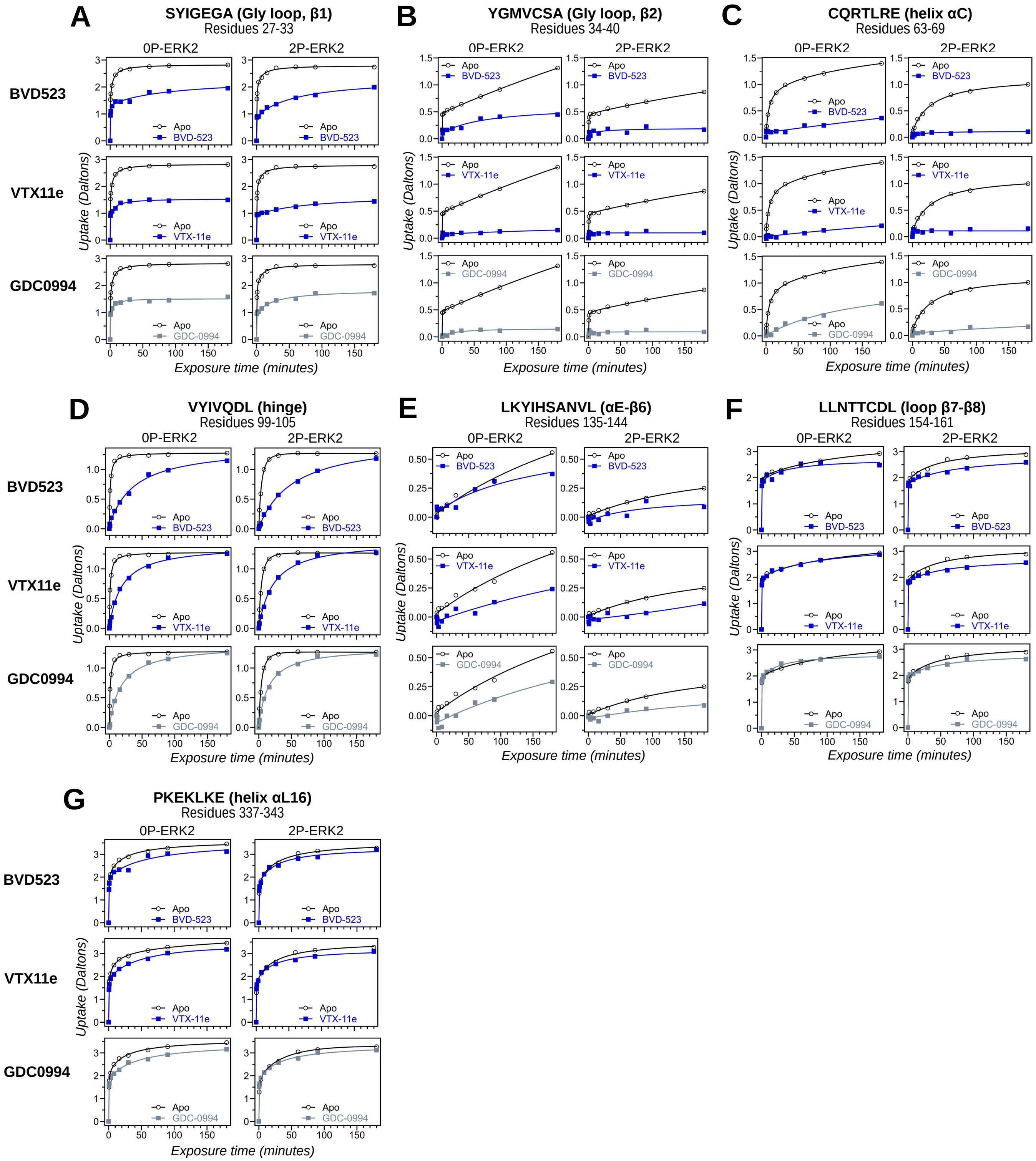

Figure S4. HDX time courses of regions with comparable responses to different inhibitors. HDX time courses for key peptide segments in ERK2, including **(A,B)** the Gly loop (peptide 29-33, SYIGEGA; peptide 34-40, YGMVCSA), **(C)** helix αC (peptide 63-69, CQRTLRE), **(D)** the hinge (peptide 99-105, VYIVQDL), **(E)** helix αE-β6 (peptide 135, 144, LKYIHSANVL), **(F)** loop β7-β8 (peptide 154-161, LLNTTCDL), and **(G)** helix αL16 (peptide 337-343, PKEKLKE). Open symbols show deuterium uptake in 0P- or 2P-ERK2 apoenzymes. Closed symbols show deuterium uptake in 0P- or 2P-ERK2 complexed with BVD523, VTX11e (blue) or GDC0994 (grey). In these regions, deuterium uptake decreases by a similar degree upon binding each of the three inhibitors.

Figure S5

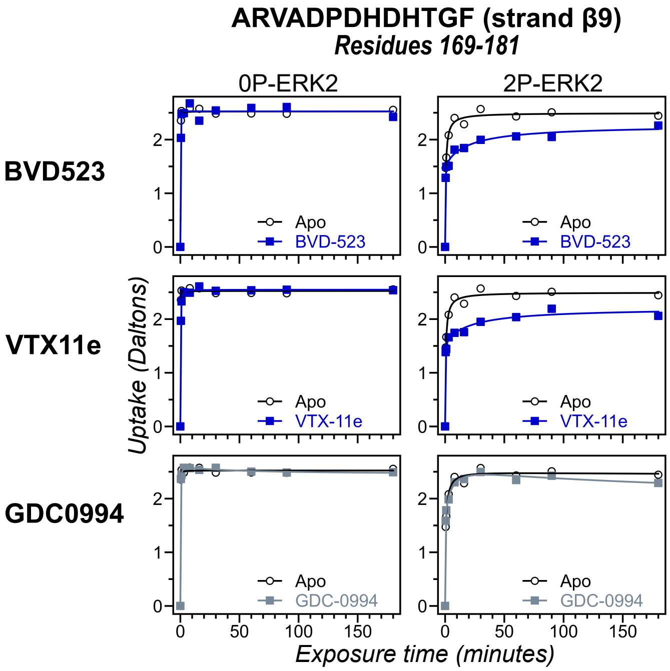

Figure S5. HDX time courses with differential responses to inhibitor binding. HDX time courses for strand β9 (peptide 169-181, ARVADPDHDHTGF) located adjacent to the DFG motif. Open symbols show deuterium uptake in 0P- or 2P-ERK2 apoenzymes. Closed symbols show deuterium uptake in 0P- or 2P-ERK2 complexed with BVD523, VTX11e or GDC0994. Deuterium uptake in these regions decreases by a larger degree upon binding VTX11e or BVD523 (blue) compared to GDC0994 (grey), as seen in the DFG motif, P+1 and helix αF (**Fig. 2**).

Figure S6A

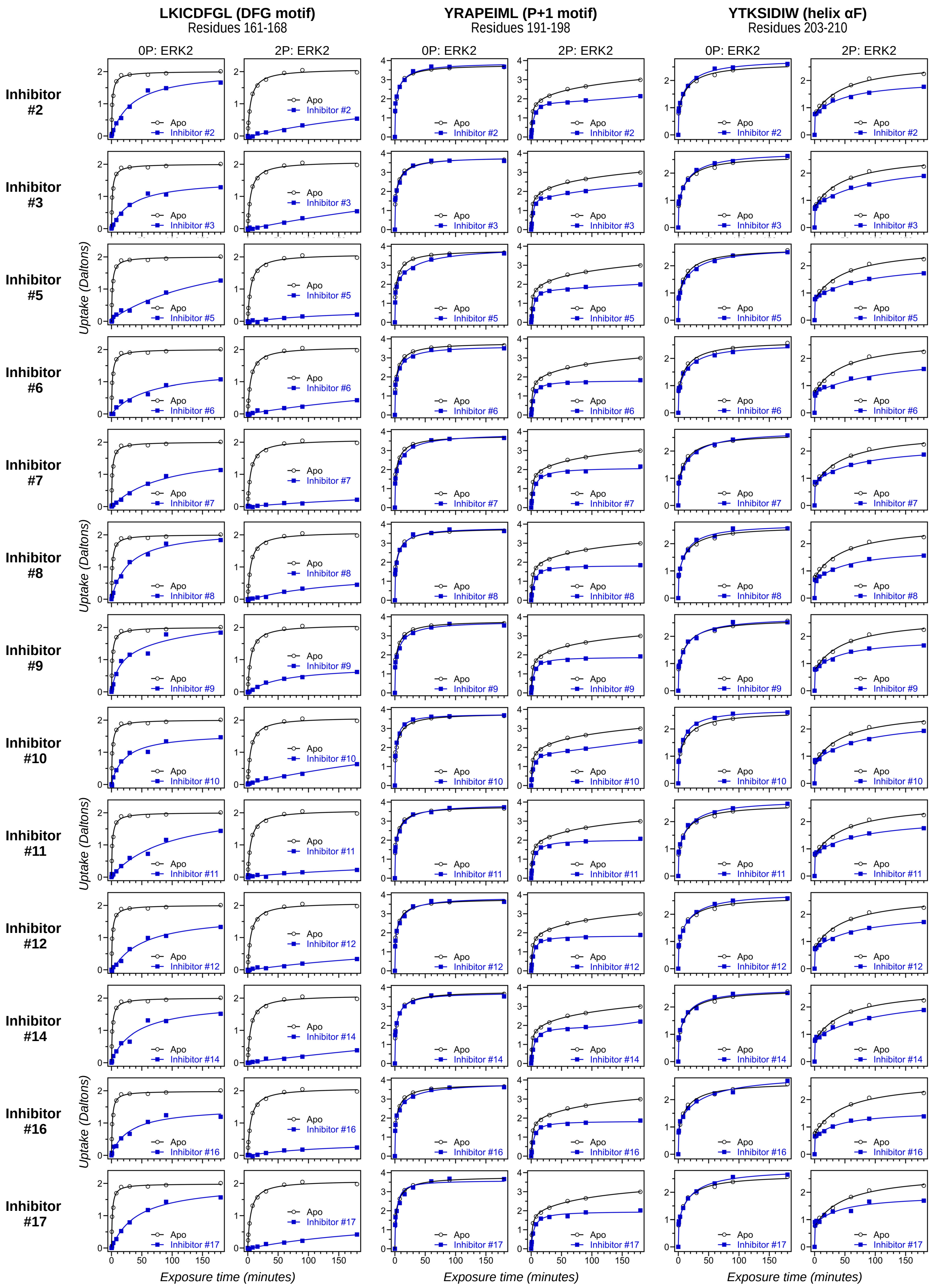

LKICDFGL (DFG motif)
Residues 161-168
YRAPEIML (P+1 motif)
Residues 191-198
YTKSIDIW (helix αF)
Residues 203-210
0P: ERK2
2P: ERK2
Inhibitor #2
Inhibitor #3
Inhibitor #5
Inhibitor #6
Inhibitor #7
Inhibitor #8
Inhibitor #9
Inhibitor #10
Inhibitor #11
Inhibitor #12
Inhibitor #14
Inhibitor #16
Inhibitor #17
Apo
Uptake (Daltons)
Exposure time (minutes)

Figure S6B

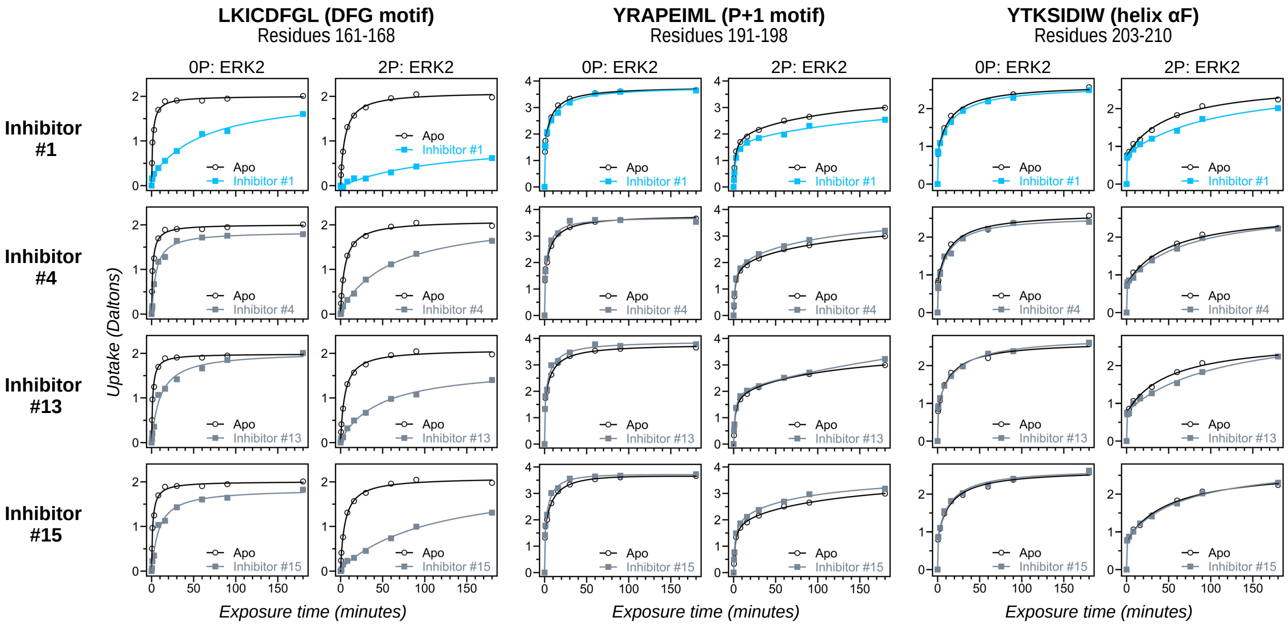

Figure S6. Differential HDX responses to new inhibitors surveyed in this study. Effects of binding the full set of inhibitors #1-#17 on deuterium uptake into the DFG motif (peptide 161-168: LKICDFGL), P+1 segment (peptide 191-198, YRAPEIML), and helix αF (peptide 203-210: YTKSIDIW), expanding the selected subset in **Fig. 4**. Open symbols show deuterium uptake in 0P- or 2P-ERK2 apoenzymes. Closed symbols show deuterium uptake in 0P- or 2P-ERK2 complexed with inhibitors. **(A)** Thirteen inhibitors (#2, #3, #5-12, #14, #16 and #17; blue) show HDX uptake patterns for 2P-ERK2 that are similar to VTX11e and BVD523, suggesting conformation selection for the R-state. **(B)** Three inhibitors (#4, #13, #15; grey) show HDX patterns similar to GDC0994, consistent with R⇌L exchange. One inhibitor (#1; cyan) shows significant HDX protection in the DFG motif, but only marginal protection in the P+1 segment or helix αF, suggesting behavior intermediate between VTX11e/BVD523 and GDC0994.

Figure S7

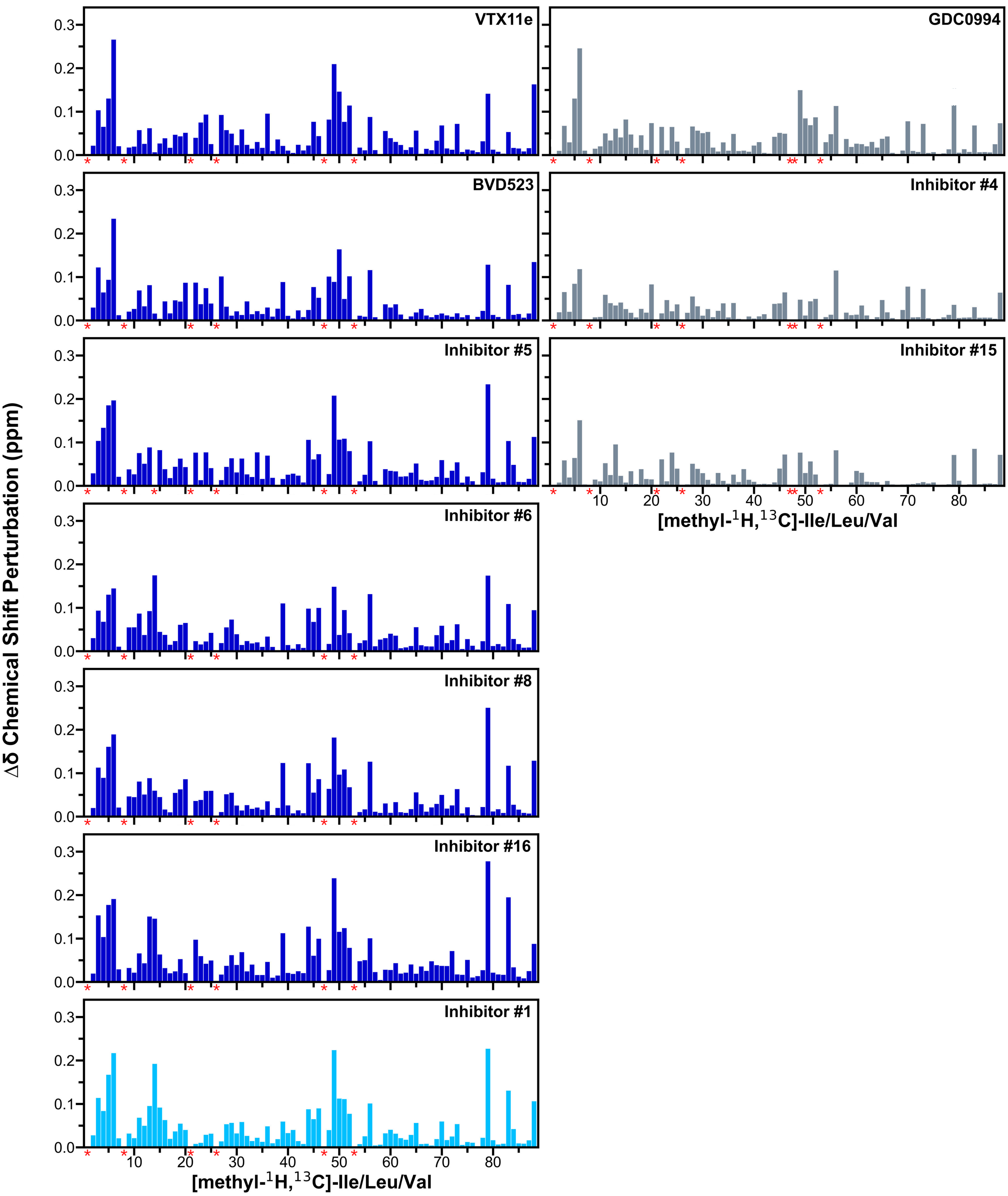

Figure S7. Chemical shift changes induced by inhibitor binding to 2P-ERK2. 2D-HMQC spectra collected on [methyl ^{1}H,^{13}C]-ILV labeled 2P-ERK2 complexed with VTX11e, BVD523, GDC0994, and the selected subset of seven inhibitors in **Fig. 6**. Chemical shift perturbations (CSP) were calculated by the equation: $\Delta\delta$ (ppm) = SQRT[(δ_H^2 + 0.20(δ_C^2)], and plotted for each ILV residue methyl. Red asterisks below the x-axes indicate methyl peaks that could not be traced in the ligand-bound state, ascribed to peak broadening. Numberings of methyl assignments, their chemical shifts and CSP calculations are listed in **Suppl. Dataset S2**.

Figure S8

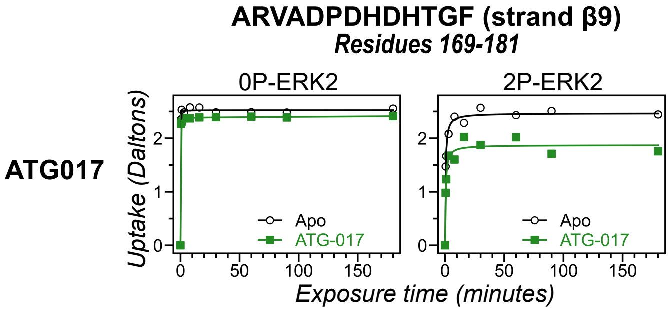

Figure S8. HDX time courses responsive to ATG017 binding. HDX time courses for strand β9 (peptide 169-181, ARVADPDHDHTGF). Open symbols show deuterium uptake in 0P- or 2P-ERK2 apoenzymes. Closed symbols show deuterium uptake in 0P- or 2P-ERK2 complexed with ATG017 (green). ATG017 decreases HDX uptake into strand β9 to a larger degree compared to GDC0994, similar to the behavior of the DFG motif, P+1 and helix αF (**Fig. 9D**).

Figure S9

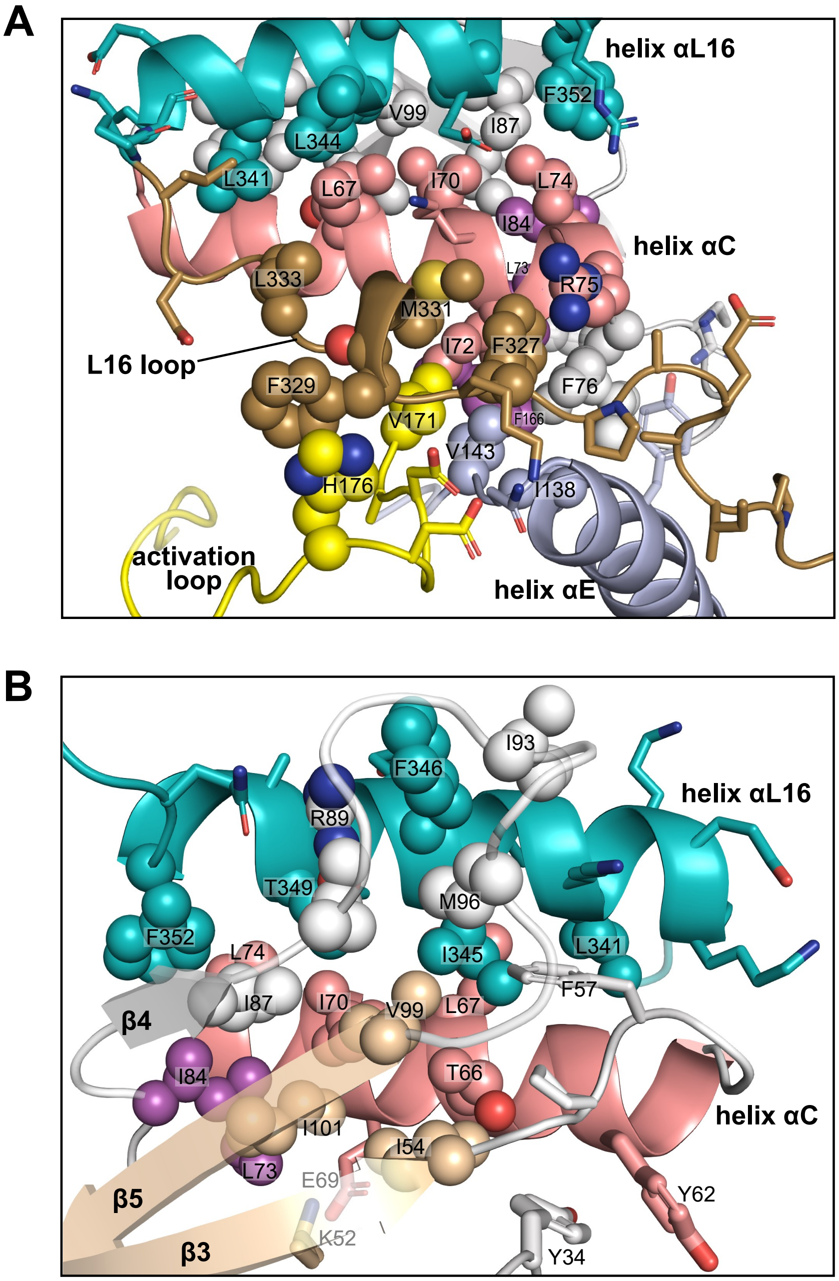

Figure S9. Interactions of L16 and helix αL16 with N-lobe elements. Views of 2P-ERK2 (PDBID:2ERK), showing hydrophobic residue interactions between **(A)** loop L16 (F327) and helices αC and αE (I72, R75, F76, I138). Residues in helix αC in turn form second sphere interactions with residues nearby catalytic residues in β3 (I54, nearby K52), β8-β9 (F166, DFG motif), and β6 (V143, nearby HRD). **(B)** Residue interactions showing close connections between helix αL16 (I345, F346, T349, F352), and helix αC (I54, F57, T66, I67, I70, L74, I87, V99) and loop β4-β5 (R89, M96,V99). Residues in magenta (L73, I84, F166) participate in the R-spine in ERK2.

Figure S10

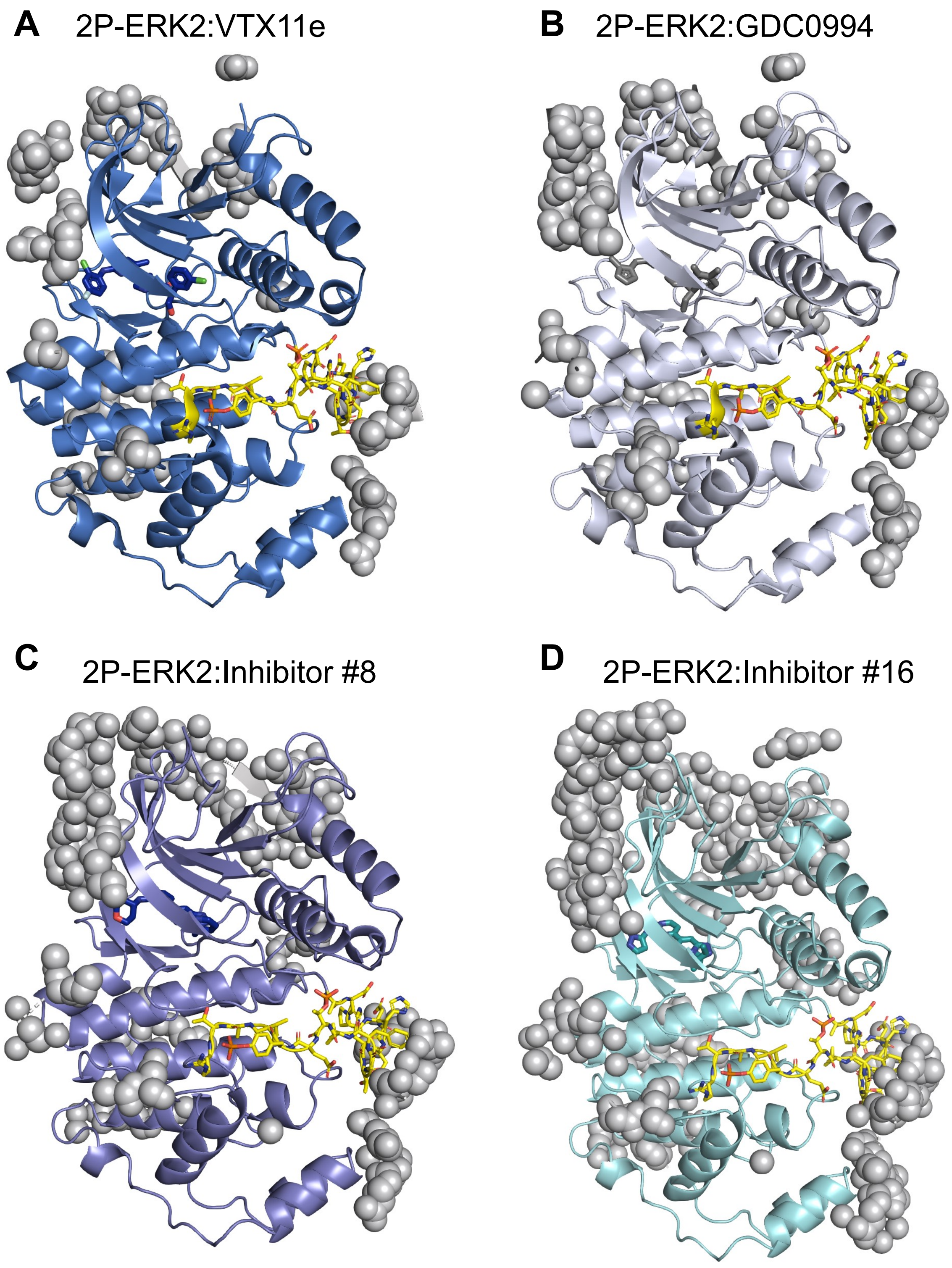

Figure S10. Crystal contacts with the activation loop in X-ray structures of ERK2. Structures of **(A)** 2P-ERK2:VTX11e (PDBID:6OPK), **(B)** 2P-ERK2:GDC0994 (PDBID:6OPH), **(C)** 2P-ERK2:Inhibitor#8, and **(D)** 2P-ERK2:Inhibitor#16 (**Suppl. Datasets S3, S4**). The activation loop is rendered in yellow, and atoms to the asymmetric unit from protein neighbors within 5 Å are shown as grey spheres.

SUPPLEMENTAL TABLE

Table S1. X-ray data collection and refinement parameters

	**2P-ERK2:Inhibitor #8**	**2P-ERK2:Inhibitor #16**
**PDB code**	TBD	TBD
**Data Collection:**		
Space group	$P2_12_12_1$	$P2_12_12_1$
Unit cell parameters (Å,°)	a = 41.98, b = 77.28,<br>c = 151.54, β = 90.0	a = 42.10, b = 76.66,<br>c = 151.97, β = 90.0
Resolution range (Å)	21.73 – 2.10<br>2.15 – 2.10	23.82 – 2.10<br>2.15 – 2.10
Number of reflections	27,964	24,273
Completeness (%)	100	100
**Refinement:**		
R_{work}/R_{free}	0.197/0.228	0.187/0.228
Total residues	556	682
Total non-hydrogen atoms	3,094	3,229
Number of protein atoms	2,858	2,873
Number of water molecules	206	329
Number of heteroatoms	30	27
RMSD bond lengths (Å)	0.010	0.009
RMSD angles (°)	1.17	1.16
Cruickshank DPI (Å)	0.18	0.21
**Average B factors ($Å^2$):**		
Average B, overall	31.23	32.56
Protein	30.88	31.61
Waters	36.70	41.29
Ligand	27.17	26.92
